# Common Femoral Artery Aneurysm: A Rare Manifestation of Immunoglobulin G4-Related Disease

**DOI:** 10.3400/avd.cr.25-00014

**Published:** 2025-04-03

**Authors:** Masaru Yoshikai, Hisashi Sato, Akito Kuwano, Naoyo Nishida

**Affiliations:** 1 Department of Cardiovascular Surgery, Shin-Koga Hospital, Kurume, Fukuoka, Japan; 2 Department of Pathology, Shin-Koga Hospital, Kurume, Fukuoka, Japan

**Keywords:** IgG4-related disease, common femoral artery, aneurysm

## Abstract

We present a case of a common femoral artery aneurysm as an extremely rare manifestation of immunoglobulin G4-related disease (IgG4-RD). A 79-year-old male, who underwent emergency abdominal aortic aneurysm repair at age 60 due to rupture, developed enlarging aneurysms of the right internal iliac artery, the right common femoral artery, and the left common femoral artery. Consequently, the right iliac artery was replaced with a graft extending to the right deep femoral artery, also reconstructing the right internal iliac artery. Histopathological examination of the resected common femoral artery wall confirmed the diagnosis of IgG4-RD.

## Introduction

Immunoglobulin G4-related disease (IgG4-RD) is a systemic inflammatory condition characterized by elevated serum IgG4 concentrations, numerous IgG4-positive lymphocytes and plasma cell infiltration, and fibrosis. Although it can affect various organs, vascular lesions in IgG4-RD predominantly manifest as periaortitis/periarteritis, with peripheral artery aneurysms being less common.^1)^ IgG4-RD involving the common femoral arteries (CFAs) is exceedingly rare, with no reports in the English literature to date.

## Case Report

**Figure 1:**
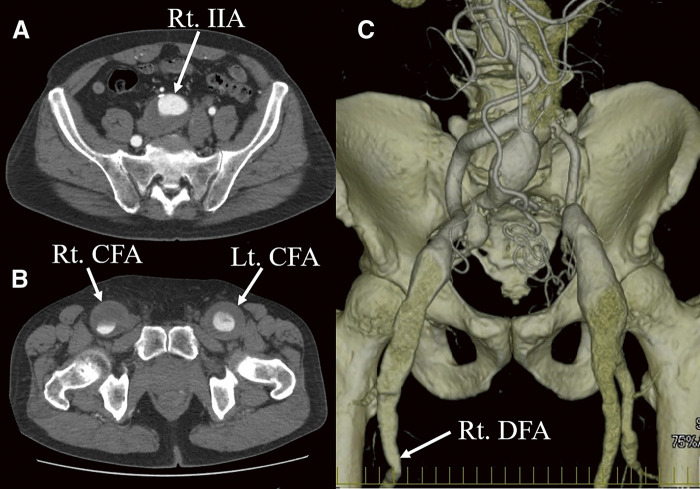
Fig. 1 Preoperative CT findings. Axial images of the abdominal CT show aneurysms of the right internal iliac artery (A) and both common femoral arteries (B), each associated with mural thrombus. Only the right deep femoral artery is opacified (C) with occlusion of the right superficial femoral artery. IIA: internal iliac artery; CFA: common femoral artery; CT: computed tomography; DFA: deep femoral artery

**Figure 2:**
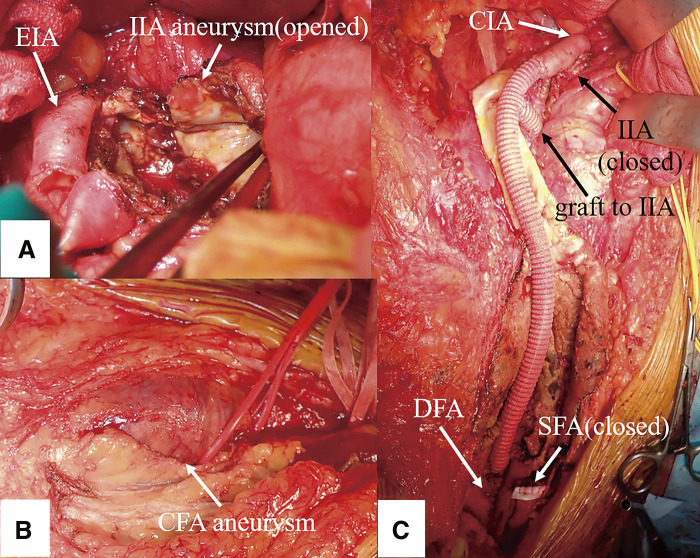
Fig. 2. Operative findings. Operative findings show aneurysms of the right internal iliac artery (A) and bilateral common femoral artery (B), and the status after the completion of procedures (C). IIA: internal iliac artery; EIA: external iliac artery; CFA: common femoral artery; CIA: common iliac artery; SFA: superficial femoral artery; DFA: deep femoral artery

**Figure 3:**
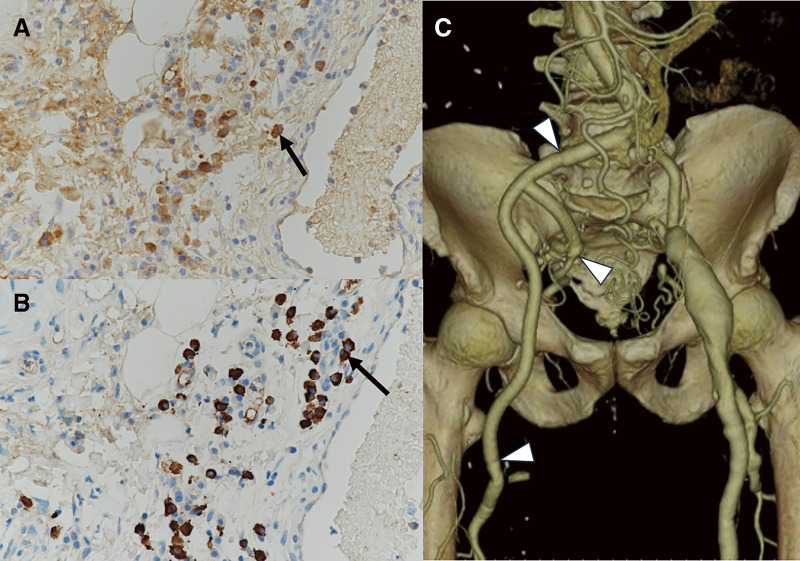
Fig. 3. Histopathological and CT findings. Histopathological study of the aneurysmal wall shows IgG-positive plasma cells (arrow in A) and IgG-4-positive plasma cells (arrow in B) (×400). (C) Postoperative 3-dimensional CT image shows excellent graft patency, with no abnormalities observed at the anastomotic sites (arrowheads). CT: computed tomography; IgG: immunoglobulin G

A 79-year-old male underwent emergency abdominal aortic aneurysm repair using a bifurcated graft at the age of 60 due to its rupture. At that time, no aneurysm was observed in the right internal iliac artery (IIA), but the right CFA was dilated to 15 mm. No histopathological examination of the abdominal aortic aneurysmal wall was performed. At age 68, he started taking warfarin and home oxygen therapy for chronic thromboembolic pulmonary hypertension, and at age 77, he underwent right lower lobe resection for hemoptysis due to dilatation of the pulmonary artery. Over the past 2 years, he experienced enlargement of the right IIA aneurysm, the right CFA aneurysm, and the left CFA aneurysm, and was referred for surgical intervention. He was being treated for hypertension, and had a history of smoking. He was asymptomatic, and pulsatile masses were palpable in both groins. The dorsalis pedis artery was palpable on the left but not on the right. Laboratory tests revealed mild renal impairment, with prothrombin-international normalized ratio (PT-INR) at 2.05, activated partial thromboplastin time (APTT) at 38.9 s, D-dimer at 41.1 μg/mL, fibrin degradation product (FDP) at 68.6 μg/dL, and C-reactive protein (CRP) level was not elevated. Ankle-brachial index was decreased at 0.65 on the right and 0.88 on the left. Echocardiography showed mild left ventricular enlargement with good left ventricular function, elevated estimated pulmonary artery systolic pressure at 52 mmHg, and mild to moderate tricuspid regurgitation. Abdominal contrast-enhanced computed tomography (CT) revealed aneurysms measuring 40 mm in the right IIA, 42 mm in the right CFA, and 44 mm in the left CFA, with thrombotic occlusion of the right superficial femoral artery (SFA) (**Fig. 1**). During the surgery, a skin incision was made parallel to the right inguinal ligament extending to the right inguinal region, and the retroperitoneal space was entered. The right limb of the prosthetic graft, the right IIA peripheral to the aneurysm, the right external iliac artery (EIA), the right SFA, and the right deep femoral artery (DFA) were exposed. The right common iliac artery was markedly adherent, making dissection difficult. Clamps were applied on the prosthetic graft, the IIA, and the DFA. The IIA aneurysm was incised and its origin was closed. A 9 mm prosthetic graft was anastomosed to the EIA and the DFA. The IIA was also reconstructed using a 9 mm prosthetic graft (**Fig. 2**). The SFA, which was thrombotically occluded, had its end closed. The anastomotic sites were wrapped with the prosthetic graft, concluding the surgery. Histopathological examination of the resected CFA aneurysmal wall revealed sclerosis with fibrosis and hyalinosis, along with rupture of elastic fibers. Infiltration of lymphocytes and plasma cells was observed in the adventitia and around the vasa vasorum. Areas with more than 10 IgG-positive plasma cells per high-power field and regions where the IgG4/IgG ratio exceeded 40% were identified (**Figs. 3A** and **3B**). However, findings of storiform fibrosis or obliterating phlebitis were not observed. Laboratory tests showed elevated IgG4 levels at 449 mg/dL, leading to a diagnosis of IgG4-RD CFA aneurysm. Postoperative contrast-enhanced CT scans confirmed good patency of the prosthetic graft (**Fig. 3C**). On postoperative day 11, the patient developed acute popliteal artery thromboembolism due to a thrombus in the left CFA aneurysm. On the same day, he experienced hemoptysis, requiring tracheal intubation and embolization of the bronchial and intercostal arteries. The ischemia and necrosis in the left lower leg worsened, necessitating limb amputation 1 month post-surgery, after which the patient was transferred to a rehabilitation hospital.

## Discussion

IgG4-RD is a systemic disease characterized by elevated serum IgG4 levels, numerous IgG4-positive lymphocytes and plasma cell infiltration, and fibrosis in affected tissues. It predominantly affects middle-aged and elderly males, and often presents with simultaneous or sequential multi-organ involvement. IgG4-RD can affect nearly all organs, and in a study of 235 cases of IgG4-RD, the most common manifestation was pancreatitis, found in 60% of cases, followed by sialadenitis, tubulointerstitial nephritis, and dacryoadenitis. Moreover, periaortitis was seen in 20% of cases, while peripheral arterial involvement, excluding aortic lesions, was rare, occurring in only 4% of cases, all of which were male.^1)^ Mizushima et al. reviewed 99 cases of IgG4-related periarteritis/retroperitoneal fibrosis, identifying perivascular lesions in 96 cases, predominantly affecting the abdominal aorta (67.7%) and iliac arteries (50.5%). Other affected sites included the coronary arteries (9.1%), the thoracic aorta (8.1%), the mesenteric artery (8.1%), and the splenic artery (2%), with single cases involving the celiac, the hepatic, the short gastric, the colonic, the femoral, the internal carotid, the vertebral, and the dural artery (1% each). Furthermore, 26.3% of cases showed luminal enlargement at the affected site, but it is not specified whether the femoral artery lesion observed in 1 case was a lumen-enlarging lesion.^2)^ Peng et al. reported that among 589 cases of IgG4-RD, 89 cases (15.2%) showed aortitis/periaortitis or periarteritis. Among the cases presenting aortitis/periaortitis or periarteritis, affected sites were predominantly the abdominal aorta (83.1%) and iliac arteries (70.8%), with aneurysms found in only 9 cases (10.1%).^3)^ A search of the English literature on PubMed for IgG4-RD involving the femoral arteries revealed only 4 reported cases of DFA aneurysms,^4–6)^ with no cases found involving the CFA. This suggests that our case is extremely rare, highlighting the unique presentation of IgG4-RD in this instance.

In IgG4-RD, the primary site of inflammation is the adventitia, which typically leads to aortitis/periaortitis and often causes thickening of the aortic and arterial walls. However, it can also lead to the formation of aneurysms in the aorta or arteries. Inoue et al.^7)^ reported a case in which the abdominal aorta was dilated, and the elastic fibers in the aortic media were disrupted due to inflammation. Similarly to the report by Inoue et al., rupture of the elastic fibers was observed in the aneurysmal wall in our case. Kasashima et al.^8)^ noted that although the relationship between the time sequence of aneurysmal formation and the development of IgG4-RD remains unclear, it is plausible to speculate that intense infiltration of inflammatory cells with the primary focus in the adventitia leads to a weakened vessel wall, which contributes to the formation of an aneurysm. This suggests that inflammation of the adventitia may extend to the media, weakening the vessel wall and resulting in the formation of aortic or arterial aneurysms.

The treatment of aortic or arterial aneurysms in IgG4-RD includes either open surgery or endovascular therapy, similar to aneurysms of other etiologies. However, for IgG4-RD abdominal aortic aneurysms, endovascular treatment has shown inferior outcomes in terms of symptom persistence, thickness of periaortic fibrosis, and serum IgG4 levels compared to open surgery. Therefore, considering complete resolution, open surgery with prosthetic graft replacement is generally preferable.^9)^ Atherosclerosis is the most common cause of true aneurysms of the femoral artery, although conditions such as Behçet’s disease, Marfan syndrome, and acromegaly may also contribute. Endovascular treatment for true aneurysms in the CFA is associated with a high frequency of complications, including thrombosis, making open surgery the standard treatment. On the other hand, conditions such as Behçet’s disease, polyarteritis nodosa, Takayasu arteritis, mycotic aneurysms, and IgG4-RD are known to cause multiple aneurysms. In this case, the patient had multiple aneurysms involving the abdominal aorta, the right IIA, and the bilateral CFAs, with no preoperative diagnosis of IgG4-RD. Given that multiple aneurysms often arise from diseases that weaken the arterial wall, there is a heightened risk of forming pseudoaneurysms at the anastomotic sites. Therefore, it is crucial to wrap the anastomotic sites with a prosthetic graft to mitigate this risk.

Steroids are the first-line treatment for IgG4-related periaortitis/periarteritis due to their effectiveness in reducing inflammation. However, in cases where luminal enlargement has occurred prior to steroid therapy, treatment with steroids can lead to thinning and weakening of periarterial lesions,^6)^ promoting arterial dilation and potentially increasing the risk of rupture.^2)^ Multivariate analysis has shown that arterial dilation prior to steroid treatment is a risk factor for further arterial enlargement after steroid therapy.^10)^ Therefore, steroid use should be cautious, especially in cases that have dilating lesions of the aorta or arteries. In the present case, since there was a left CFA aneurysm, steroid treatment was not initiated, and treatment options for this aneurysm are currently under consideration.

## Conclusions

IgG4-RD involving the CFA aneurysm is extremely rare. This case also presented with multiple aneurysms, suggesting a potential underlying disease which causes fragility of the vascular wall. Therefore, to prevent pseudoaneurysm formation at the anastomotic site, a prosthetic graft was used to wrap the anastomotic sites. Due to the presence of a left CFA aneurysm, steroid treatment was not initiated due to concerns about the potential dilatation of the aneurysm.

## Declarations

### Informed consent

Written informed consent was obtained from the patient to publish this case report.

### Ethics approval

We obtained approval from the institutional ethics committee of Shin-Koga Hospital (Approval number: 24-0054).

### Disclosure statement

The authors declare that they have no conflicts of interest.

### Author contributions

Study conception: MY

Data collection: MY

Analysis: MY

Manuscript preparation: MY

Critical review and revision: all authors

Final approval of the article: all authors

Accountability for all aspects of the work: all authors.
